# Association Between High-Intensity Knee Extensor Torque Steadiness and Center-of-Pressure Path Length During a Backward Reach-Hold Task

**DOI:** 10.7759/cureus.115607

**Published:** 2026-09-01

**Authors:** Atsushi Ueda, Yasuhiro Mitani, Hitoshi Koda, Toshimitsu Omine, Mizuki Nakamura, Ryuta Inada

**Affiliations:** 1 Department of Rehabilitation Sciences, Faculty of Allied Health Sciences, Kansai University of Welfare Sciences, Kashiwara, JPN; 2 Faculty of Arts and Sciences, Kyoto Institute of Technology, Kyoto, JPN; 3 Department of Rehabilitation, Shimada Hospital, Habikino, JPN

**Keywords:** center of pressure, knee extensors, postural behavior, reach-hold task, single-leg stance, torque steadiness

## Abstract

Introduction: Quadriceps function contributes to lower-extremity support during single-limb tasks. It can be characterized by maximal voluntary contraction (MVC) and submaximal torque steadiness. MVC reflects maximal knee extensor torque-generating capacity, whereas torque steadiness reflects the ability to regulate submaximal torque output. These aspects of quadriceps function may contribute to postural regulation during single-limb balance tasks, including lower-extremity reach tasks. However, their relationships with center of pressure (COP) behavior during reach-hold tasks remain unclear.

Methods: A total of 32 healthy young adults performed quiet single-leg stance, forward reach-hold, and backward reach-hold tasks. Anteroposterior (AP) and mediolateral (ML) COP path lengths were recorded. Knee extensor MVC and torque steadiness at 10%, 40%, and 70% MVC were assessed. Torque steadiness was quantified using the coefficient of variation from the most stable five-second window within each 15-second trial. Associations between knee extensor variables and task-specific COP measures were examined using Pearson’s or Spearman’s correlation coefficients, as appropriate. The Holm procedure was applied to adjust for multiple correlation tests. A supplementary simple linear regression analysis was performed for the association that remained significant after Holm correction.

Results: Compared with single-leg stance, AP COP path length was greater during forward and backward reach-hold, whereas ML COP path length was greater during backward reach-hold. In the unadjusted analyses, normalized MVC torque and torque CV at 40% MVC were moderately associated with AP COP path length during forward reach-hold. Torque CV at 70% MVC was also moderately associated with AP and ML COP path lengths during backward reach-hold. After Holm correction, only the positive association between torque CV at 70% MVC and AP COP path length during backward reach-hold remained statistically significant (ρ = 0.48, 95% CI (0.14, 0.71), unadjusted *p* = 0.006, Holm-adjusted* p* = 0.035). Greater high-intensity torque variability was therefore associated with a longer AP COP path. In the supplementary regression analysis, torque CV at 70% MVC explained 18.9% of the variance in AP COP path length (R² = 0.189, *p* = 0.013).

Conclusions: Reduced knee extensor torque steadiness at high intensity was associated with greater AP COP path length during backward reach-hold in healthy young adults. Associations involving maximal torque capacity, moderate-intensity torque steadiness, and ML COP behavior were observed only in the unadjusted analyses and should be regarded as exploratory. These findings suggest that high-intensity torque regulation may be relevant to AP postural behavior during a demanding backward reach-hold task; however, confirmation in larger and clinical populations is required.

## Introduction

Lower-extremity musculoskeletal injuries or disorders, including anterior cruciate ligament injury or reconstruction, patellofemoral pain, and meniscal injury, frequently result in reduced performance in daily and sport-related activities [1-3]. These functional limitations are commonly accompanied by impairments in static and dynamic balance [4-6], as well as quadriceps weakness or other deficits in quadriceps function [1,6,7]. Quadriceps dysfunction may be associated with reduced stance-limb support, impaired control of knee flexion, and altered regulation of lower-extremity loading during weight-bearing activities [5,6,8,9]. Because movement performance is influenced by multiple neuromuscular and biomechanical factors, quadriceps dysfunction should not be considered the sole determinant of balance impairment. Nevertheless, clarifying the relationship between quadriceps function and lower-extremity balance behavior may improve understanding of the functional factors underlying movement limitations and may help complement clinical assessment and rehabilitation planning.

Quadriceps function is commonly assessed using maximal voluntary contraction (MVC), which represents maximal knee extensor torque-generating capacity. Although maximal strength is clinically important, most postural, daily, and sport-related tasks are performed at submaximal force levels and require continuous adjustment of torque output as body position and task demands change. Torque steadiness provides an index of the consistency of torque output around a prescribed submaximal target and is commonly quantified using the coefficient of variation during a sustained contraction [10-12]. Thus, MVC and torque steadiness represent different aspects of quadriceps function: MVC reflects maximal torque capacity, whereas torque steadiness reflects the consistency of submaximal torque regulation. Evaluating both measures may therefore provide a more comprehensive characterization of quadriceps function than maximal strength testing alone.

Previous studies have examined the relationship between muscle force steadiness and postural sway and have reported associations between variability in submaximal force output and postural behavior during quiet and single-leg standing. In particular, low-intensity force steadiness of the plantar flexors has been associated with postural sway during relatively low-demand standing tasks, suggesting that the ability to regulate small levels of muscle force consistently may contribute to maintaining upright posture [13,14]. Associations between knee extensor force control and balance performance have also been reported [15]. However, most previous evidence has focused on relatively low-intensity force control and lower-demand postural tasks, whereas it remains unclear whether the relationships of knee extensor torque steadiness with postural behavior differ between quiet single-leg stances and more demanding unilateral tasks that require the body to be displaced while the stance knee remains flexed. The knee extensors contribute to controlling knee flexion, supporting the body, and regulating lower-extremity loading during weight-bearing tasks. Therefore, maximal knee extensor torque capacity and submaximal torque regulation may show different relationships with postural behavior during quiet single-leg stances and during more demanding single-limb tasks performed with the knee in a flexed position. Moreover, the functional relevance of torque steadiness may vary according to both postural task demand and contraction intensity. Assessment at a single submaximal intensity may not capture potential intensity-related differences because torque regulation at low, moderate, and high target intensities may impose different demands on neuromuscular control. Therefore, based on previous studies [10,15], torque steadiness was assessed at 10%, 40%, and 70% MVC to represent low-, moderate-, and high-intensity conditions, respectively. Assessing torque steadiness across these three intensities permits comparison of force-regulation characteristics across a broad range of submaximal demands that may not be captured by maximal strength testing or testing at a single submaximal intensity. These intensity categories were used as practical testing conditions and were not intended to represent established physiological or clinical thresholds. However, it remains unclear whether lower-extremity postural behavior is associated with maximal torque capacity, submaximal torque steadiness, or both, and which of these submaximal intensity conditions is most relevant during demanding single-limb tasks.

Single-leg stance and reach tests are widely used to assess lower-extremity balance in orthopedic, sports medicine, and rehabilitation settings [5,16-18]. Compared with quiet single-leg stances, forward and backward reach tasks impose greater stance-limb support and postural regulation demands because participants must maintain unilateral support while moving and holding the body relative to the stance foot. These sagittally directed tasks may be particularly relevant to knee extensor function because the stance knee remains flexed while the reach position is maintained. Under these conditions, the quadriceps may contribute to stance-limb support, regulation of knee flexion, and control of lower-extremity loading. Forward and backward reaching involve different displacements of the body relative to the stance foot and may consequently require different combinations of lower-extremity and trunk control. Including both directions therefore enables examination of whether the associations of maximal torque capacity and submaximal torque steadiness with center of pressure (COP) behavior are consistent across, or vary between, distinct postural-control demands. The purpose of comparing the two reach directions was not merely to confirm expected directional differences in COP behavior, but to examine whether the functional relevance of knee extensor capacity and torque regulation depended on the demands imposed by the reach direction. However, forward and backward reach tasks should not be regarded as direct measures of quadriceps function, because ankle, hip, trunk, and sensory factors also contribute substantially to task performance. Rather, they provide functionally relevant conditions under which the contribution of knee extensor capacity and torque regulation can be examined.

Reach-based balance assessments are commonly quantified using maximal reach distance [5,16,19]. Although reach distance is a practical measure of overall task performance, it is an endpoint outcome and may not fully characterize how the stance limb behaves while the target position is maintained. Individuals may achieve similar reach distances using different combinations of trunk, hip, knee, ankle, and foot strategies [20,21]. COP assessment during a controlled reach-hold phase may therefore complement reach distance by quantifying postural behavior beneath the stance foot while the target position is maintained. Examining COP during reach-hold tasks may help determine whether maximal knee extensor capacity and submaximal torque regulation are associated with task-specific postural behavior beyond reach performance alone.

Therefore, this study examined whether the associations of knee extensor MVC and torque steadiness at 10%, 40%, and 70% MVC with COP behavior differed among quiet single-leg stance, forward reach-hold, and backward reach-hold tasks in healthy young adults. We hypothesized that COP path length would be greater during the reach-hold tasks than during quiet single-leg stances and that COP path length during the reach-hold tasks would be associated with knee extensor torque steadiness.

## Materials and methods

Participants

This cross-sectional study included 32 healthy young adults (18 male and 14 female participants). Participants were excluded if they had a history of lower limb or lumbar musculoskeletal disorders or surgery. The study was approved by the Ethics Committee of Kansai University of Welfare Sciences (approval number: 25-18); written informed consent was obtained from all participants.

An a priori sample size calculation was conducted using G*Power 3.1 (Heinrich-Heine-Universität Düsseldorf, Düsseldorf, Germany) for two-tailed correlation analyses (α = 0.05, power = 0.80). Based on an expected association of r = 0.45 from previous literature (r ≈ 0.35-0.45), the required sample size was 26 participants [14]. To account for potentially unusable trials, 32 participants were recruited.

The dominant leg (defined as the preferred kicking leg) was used for all measurements. Anthropometric variables included height, body mass, ankle range of motion (ROM) (dorsiflexion and plantarflexion), knee ROM (flexion and extension), lower limb length (LLL) (symphysis to medial malleolus; SMD), and foot length.

Experimental tasks and center of pressure analysis

Participants performed three single-leg support tasks on the dominant stance limb: single-leg stance, forward reach-hold, and backward reach-hold. For reach-hold tasks, the contralateral limb was positioned at a predefined target distance in the forward or backward direction, and the end position was maintained while balance was sustained on the stance limb. Task order was randomized. Each trial lasted 35 s, and the middle 30 s were analyzed to minimize transient effects. Two trials were performed for each task, with 30 s rest between trials and 1 min between tasks. Participants completed standardized warm-up and practice trials prior to data collection.

Reach distances were standardized relative to LLL, with forward reach set at 60% LLL and backward reach at 100% LLL. These values were selected based on pilot testing to ensure that participants could maintain the required hold duration without compromising task feasibility and were informed by previous reports indicating maximal forward and backward reach distances of approximately 70% and 110% of LLL in healthy young adults [15]. All balance and reach-hold tasks were performed barefoot to standardize the foot-support surface interface across participants. During reach-hold tasks, the contralateral (reaching) limb was not used for weight-bearing support. Participants reached toward a lightweight block or floor marker and were permitted light toe contact only as a positional or tactile cue, without intentional loading. Trials were repeated if weight-bearing through the reaching limb was observed or if movement of the stance foot occurred.

COP was recorded using a force plate (Type 9286BA; Kistler Group, Winterthur, Switzerland) at a sampling frequency of 1,000 Hz. COP data were expressed in a laboratory-fixed coordinate system (X: ML; Y: AP) and low-pass filtered using a fourth-order Butterworth filter with a 10 Hz cutoff frequency. Total path length and mean velocity were calculated separately for the ML and AP directions over the 30-s analysis window. COP processing was performed using MATLAB (MathWorks, Natick, MA).

Knee extension torque measurement and analysis

Knee extensor torque was measured using an isokinetic dynamometer (Biodex System 3 dynamometer; Biodex Medical Systems, Shirley, NY). Participants were seated with the hip flexed to approximately 85° and the knee fixed at 60° of flexion. Following a standardized warm-up and familiarization, maximal MVC of the quadriceps was assessed.

For MVC testing, participants were instructed to exert maximal effort for 5 s. Two MVC trials were performed with 30 s of rest between trials. MVC torque was defined as the mean of the two trials and normalized to body weight (%BW) to reduce inter-individual differences related to body size. Torque steadiness was assessed at 10%, 40%, and 70% of MVC. Participants maintained the target torque for 15 s with real-time visual feedback. After one familiarization trial, two trials were performed at each intensity, with 30 s rest between trials and 1 min rest between intensities; testing order was randomized. Torque steadiness was quantified using the coefficient of variation (CV = standard deviation/mean × 100). The primary analysis used a sliding window approach to identify the 5-s interval with the lowest SD within each 15-s trial (minSD-5s) from which CV was calculated [22]. All torque analyses were performed using MATLAB (MathWorks, Natick, MA, USA).

Statistical analysis

All statistical analyses were performed using SPSS Statistics version 29 (IBM Corp., Armonk, NY). The normality of the distributions was assessed using the Shapiro-Wilk test. Torque CV at 10%, 40%, and 70% MVC showed significant deviations from normality (p < 0.05), whereas no significant deviation from normality was detected for the other variables (p > 0.05).

To investigate task-dependent differences in COP measures, separate one-way repeated-measures ANOVAs were performed for AP and ML COP path lengths across the three task conditions. When the assumption of sphericity was violated, the Greenhouse-Geisser correction was applied. Bonferroni-adjusted post hoc comparisons were performed, and partial eta squared (ηp²) was reported as the effect size. For descriptive interpretation, ηp² values of 0.01, 0.06, and 0.14 were considered indicative of small, medium, and large effects, respectively. These thresholds were used as general benchmarks rather than definitive or context-independent classifications.

For the association analyses, correlations were computed between knee extensor MVC torque or torque steadiness (CV at 10%, 40%, and 70% MVC) and COP outcomes (AP and ML path lengths) separately for the forward and backward reach-hold tasks. Correlation analyses were not performed for the quiet single-leg stance because the primary purpose of these analyses was to examine the associations of knee extensor capacity and torque regulation with COP behavior during the more demanding reach-hold tasks. Pearson’s product-moment correlations were used for associations involving MVC torque because no significant deviation from normality was detected for the relevant variables. Spearman’s rank correlations were used for associations involving torque CV because the CV variables were not normally distributed. Correlation magnitudes were interpreted as trivial (<0.10), small (0.10-0.29), moderate (0.30-0.49), large (0.50-0.69), very large (0.70-0.89), and nearly perfect (≥0.90) [23].

To control the family-wise error rate arising from multiple correlation tests, the Holm procedure was applied separately within each reach direction. Within each direction, the four correlations between MVC torque and the AP and ML COP outcomes were treated as one family, whereas the six correlations between torque CV at the three intensities and the two COP outcomes were treated as a separate family. Both unadjusted and Holm-adjusted p-values are reported. Statistical significance in the correlation analyses was determined using the Holm-adjusted p-values. The correlation analyses were considered hypothesis-generating, and their interpretation was based on the magnitude and precision of the correlation estimates, as indicated by the correlation coefficients and their 95% confidence intervals, together with Holm-adjusted statistical significance.

When an association remained statistically significant after Holm correction, simple linear regression was performed as a supplementary analysis to estimate the explained variance (R²). The assumptions of linearity, homoscedasticity, normality of residuals, and absence of influential outliers were assessed before interpreting the regression model. Regression results were interpreted as the proportion of variance in the COP outcome explained by the predictor and not as evidence of a causal or prospectively predictive relationship. For analyses other than the correlation analyses, a two-sided p-value of <0.05 was considered statistically significant.

## Results

The results are summarized in Tables 1-2.

**Table 1:** Participants’ demographic data SD, standard deviation

Demographic data	Mean	SD
Age, year	20.21	0.55
Height, cm	164.92	8.86
Body weight, kg	61.79	16.46
Lower-limb length, cm	81.32	5.29
Foot length, cm	23.50	4.45
Maximum lower-limb reach distance forward, % leg length	73.78	7.82
Maximum lower-limb reach distance backward, % leg length	117.87	7.64

**Table 2:** Knee extension maximal torque and torque steadiness MVC, maximum voluntary contraction; CV, coefficient of variation. ^a^ Mean, standard deviation; ^b^ Median, interquartile range.

Torque parameters	Values	Measures of variability
MVC torque, Nm/kg^a^	2.66	0.59
CV at 10% MVC, %^b^	1.94	1.59
CV at 40% MVC, %	1.28	0.60
CV at 70% MVC, %	1.64	0.95

COP path length differed significantly among the three postural tasks (Figure 1). Repeated-measures analysis of variance revealed significant main effects of task for both ML and AP COP path lengths (ML: F(1.62, 50.27) = 18.20, p < 0.001, partial η² = 0.37; AP: F(1.39, 43.00) = 10.60, p < 0.001, partial η² = 0.26). ML COP path length increased progressively from single-leg stance to forward reach-hold and backward reach-hold, with significant differences among all three conditions (all p < 0.05). AP COP path length was greater during both forward and backward reach-hold than during single-leg stance (both p < 0.01), whereas no significant difference was observed between the two reach-hold conditions (p = 1.000).

**Figure 1:**
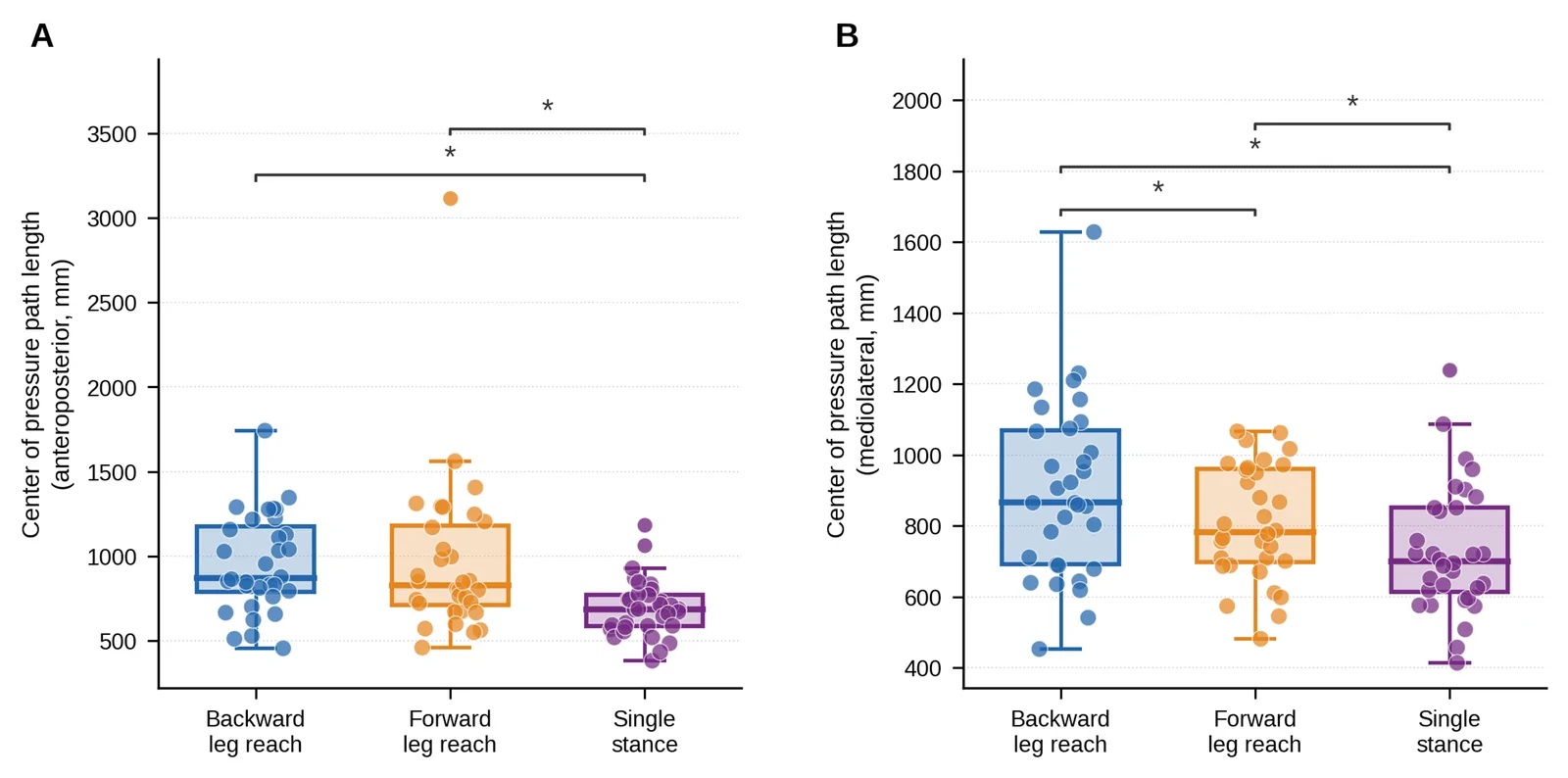
Task-dependent differences in center of pressure measures during single-leg balance tasks Box-plots show anteroposterior and mediolateral center of pressure path length (mm) during backward reach-hold, forward reach-hold, and single-leg stance. Each point represents an individual participant. Horizontal bars with an asterisk indicate significant pairwise differences after Bonferroni adjustment (*p < 0.05).

The associations between knee extensor variables and COP measures during the forward and backward reach-hold tasks are summarized in Tables 2-3. During forward reach-hold, normalized MVC torque (r = 0.36, 95% CI (0.01, 0.63), unadjusted p = 0.04) and torque CV at 40% MVC (ρ = 0.39, 95% CI (0.04, 0.66), unadjusted p = 0.027) were positively associated with AP COP path length in the unadjusted analyses; however, neither association remained statistically significant after Holm correction (Holm-adjusted p = 0.17 and 0.16, respectively). During backward reach-hold, torque CV at 70% MVC was positively associated with both AP (ρ = 0.48, 95% CI (0.14, 0.71), unadjusted p = 0.006) and ML COP path lengths (ρ = 0.38, 95% CI (0.03, 0.65), unadjusted p = 0.03). After Holm correction, only the association with AP COP path length remained statistically significant (Holm-adjusted p = 0.035; Figure 2), whereas the association with ML COP path length did not (Holm-adjusted p = 0.15). Thus, the only association that remained statistically significant after Holm correction was the positive association between torque CV at 70% MVC and AP COP path length during backward reach-hold. A supplementary simple linear regression analysis showed that torque CV at 70% MVC explained 19% of the variance in AP COP path length during backward reach-hold (R² = 0.19, p = 0.013).

**Figure 2:**
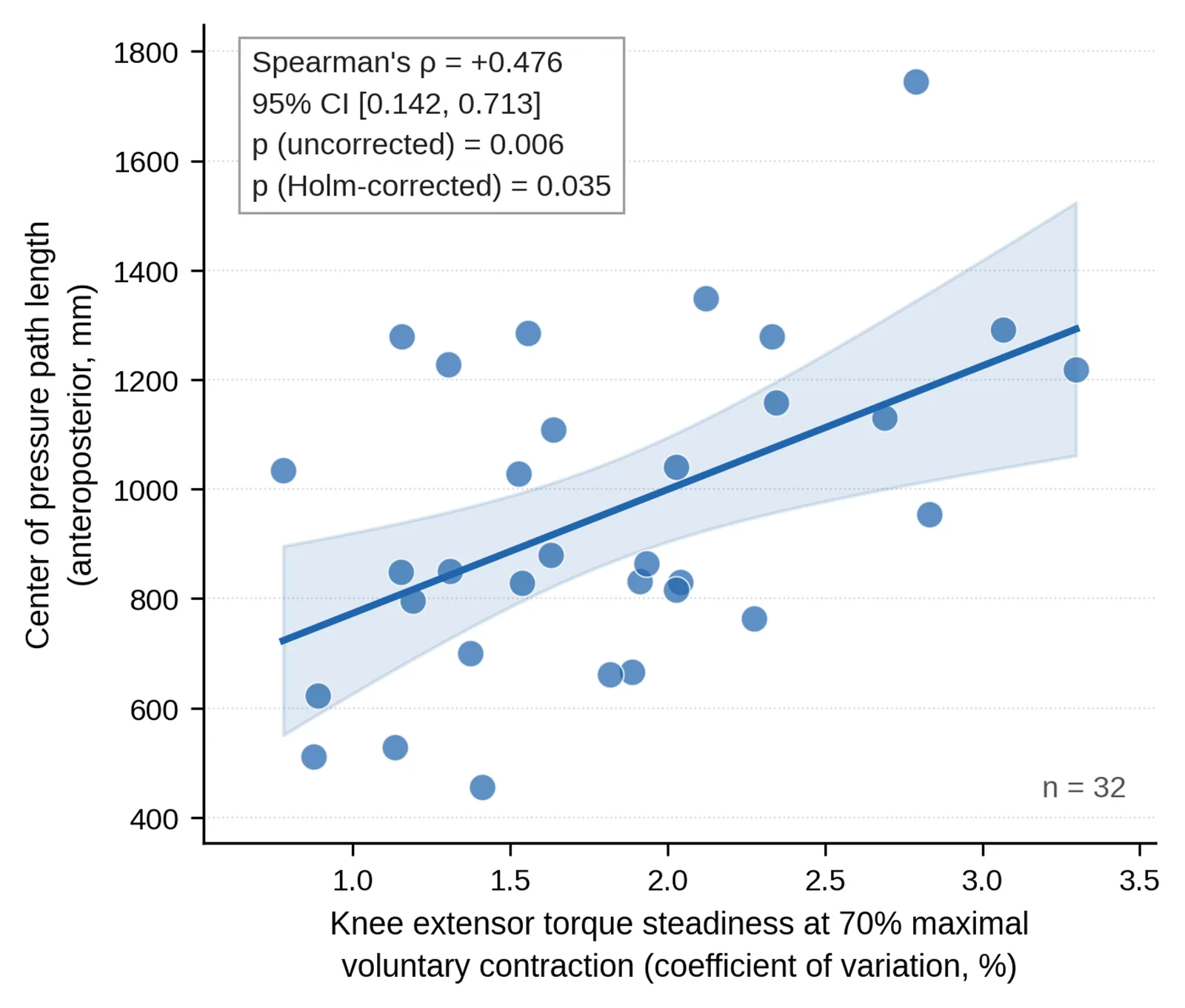
Significant associations between knee extensor torque steadiness and center of pressure path length during the backward reach-hold task Association between knee extensor torque variability at 70% of maximum voluntary contraction and anteroposterior center-of-pressure path length during the backward reach-hold task. Torque variability was expressed as the coefficient of variation. The association was evaluated using Spearman’s rank correlation coefficient (ρ = 0.48, 95% CI (0.14, 0.71), unadjusted p = 0.006, Holm-adjusted p = 0.035).

**Table 3:** Correlation between center of pressure parameters and knee extension torque during single-leg support tasks Values are presented as Pearson’s correlation coefficients (r) for MVC torque and Spearman’s rank correlation coefficients (ρ) for torque CV. Unadjusted and Holm-adjusted p values are reported. Holm adjustment was applied across the four correlations involving MVC torque and separately to the six correlations involving torque CV within each reach direction. Asterisks indicate statistical significance after Holm correction (p-adjusted < 0.05). 95% CI, confidence interval; ML, mediolateral; AP, anteroposterior; COP, center of pressure; MVC, maximum voluntary contraction; CV, coefficient of variation.

A. Backward leg reach	ML path length					AP path length				
	Coefficient	95% CI		Unadjusted p	Holm-adjusted p	Coefficient	95% CI		Unadjusted p	Holm-adjusted p
		Lower limit	Upper limit				Lower limit	Upper limit		
MVC torque, Nm/kg	0.15	-0.21	0.47	0.426	0.852	0.30	-0.05	0.59	0.09	0.281
CV at 10% MVC, %	0.12	-0.25	0.46	0.525	1.000	-0.06	-0.41	0.30	0.74	1.000
CV at 40% MVC, %	0.31	-0.05	0.60	0.084	0.338	0.24	-0.13	0.55	0.19	0.555
CV at 70% MVC, %	0.38	0.03	0.65	0.030	0.151	0.48	0.14	0.71	0.01	0.035*
B. Forward leg reach	ML path length					AP path length				
	Coefficient	95%CI		Unadjusted p	Holm-adjusted p	Coefficient	95%CI		Unadjusted p	Holm-adjusted p
		Lower limit	Upper limit				Lower limit	Upper limit		
MVC torque, Nm/kg	0.05	-0.31	0.39	0.798	0.852	0.36	0.01	0.63	0.042	0.169
CV at 10% MVC, %	0.14	-0.23	0.48	0.441	1.000	0.02	-0.34	0.38	0.895	1.000
CV at 40% MVC, %	0.32	-0.04	0.61	0.071	0.354	0.39	0.04	0.66	0.027	0.163
CV at 70% MVC, %	0.17	-0.21	0.49	0.368	1.000	0.24	-0.13	0.55	0.193	0.773

## Discussion

The principal finding of this study was that greater knee extensor torque variability at 70% MVC was moderately associated with a longer AP COP path during backward reach-hold, and this association remained statistically significant after Holm correction. In contrast, the associations of normalized MVC torque and torque CV at 40% MVC with AP COP path length during forward reach-hold, as well as the association of torque CV at 70% MVC with ML COP path length during backward reach-hold, did not remain significant after correction and should therefore be regarded as exploratory. Collectively, these findings provide preliminary evidence that knee extensor torque regulation at the relatively high testing intensity of 70% MVC may be related to AP COP behavior during backward reach-hold; however, they do not establish consistent direction- or intensity-specific relationships across the two reach-hold conditions examined.

COP behavior also differed across the three postural tasks. COP path length is a linear stabilometric descriptor representing the total distance traveled by the COP during the analysis period and should not be interpreted as a direct measure of better or poorer postural control [18,24]. Therefore, the greater COP path lengths observed in this study indicate that more COP movement occurred while participants maintained the task position. Compared with quiet single-leg stance, both forward and backward reach-hold produced greater AP and ML COP path lengths. Moreover, backward reach-hold produced a greater ML COP path length than forward reach-hold, whereas AP COP path length did not differ significantly between the two reach-hold tasks. These patterns suggest that the two reach directions may have imposed different postural-control demands rather than simply differing in overall task difficulty. The greater ML COP path length during backward than forward reach-hold may indicate that backward reach-hold involved greater mediolateral regulation. However, because lower-extremity and trunk kinematics were not recorded, the specific movement strategies responsible for these COP patterns cannot be determined. Previous studies of the Star Excursion Balance Test and Y-Balance Test have demonstrated that reach performance involves coordinated changes in lower-extremity and trunk positions and that joint kinematics, kinetics, and muscle activation vary according to reach direction [20,25-27]. Although the underlying movement strategies were not measured, the present COP differences are consistent with the involvement of direction-dependent adjustments of the trunk, pelvis, hip, knee, and ankle during unilateral support.

The association between torque CV at 70% MVC and AP COP path length during backward reach-hold was the only association that remained statistically significant after correction for multiple testing. Because torque steadiness was quantified using the coefficient of variation, the positive association indicates that greater high-intensity torque variability, representing poorer steadiness, was associated with a longer AP COP path. Torque steadiness is influenced by contraction intensity, joint position, and the mechanical conditions under which torque is produced [28-30]. Thus, torque-regulation characteristics measured under a relatively high submaximal load may be relevant to maintaining a displaced body position during the backward reach-hold task. However, the 70% MVC testing condition should not be interpreted as representing the actual quadriceps activation or loading level during backward reach-hold. Moreover, because electromyographic activity, joint kinematics, and joint moments were not recorded, the present data cannot identify the neuromuscular or biomechanical mechanism underlying this association. The finding therefore supports an association between laboratory-measured torque variability at a relatively high testing intensity and AP COP movement during backward reach-hold, rather than a direct mechanistic relationship between quadriceps activation and COP path length.

The associations observed only in the unadjusted analyses require more cautious interpretation. During forward reach-hold, normalized MVC torque and torque CV at 40% MVC showed moderate positive correlations with AP COP path length, whereas torque CV at 70% MVC showed a moderate correlation with ML COP path length during backward reach-hold. None of these associations remained statistically significant after Holm correction. In particular, the unadjusted association involving torque CV at 40% MVC does not provide sufficient evidence for a selective moderate-intensity mechanism during forward reach-hold, nor does the absence of statistically significant associations at 10% and 70% MVC demonstrate that the correlation differed significantly among intensities. These patterns should therefore be considered hypothesis-generating and require independent replication. Nevertheless, because a corrected association was identified in only one task, at one intensity, and in one COP direction, and because a Holm-adjusted association was identified in only one reach condition, at one contraction intensity, and in one COP direction, the present findings do not establish that the strength of the association differed between reach directions, contraction intensities, or COP directions.

The clinical implications are consequently limited. In the supplementary simple linear regression analysis, torque CV at 70% MVC accounted for 18.9% of the variance in AP COP path length during backward reach-hold (R² = 0.189). This represents a modest proportion of the variance, indicating that most of the interindividual variation in AP COP path length was associated with other measured or unmeasured factors. COP movement reflects the combined contributions of ankle and hip function, trunk strategy, sensory integration, foot-pressure distribution, task familiarity, and other individual characteristics [20,25-27]. Torque steadiness may eventually complement MVC by providing information about submaximal torque regulation, but the present results do not establish the clinical utility of combining torque steadiness with reach-hold COP assessment. In particular, these measures should not replace assessments of symptoms, joint function, reach performance, movement strategy, or activity-specific capacity.

Several limitations should be acknowledged. First, the relatively small sample consisted exclusively of healthy young adults recruited from a single institution. These sampling characteristics limit generalizability to other settings, older adults, athletes following injury, and patients with knee disorders. Second, the cross-sectional design does not permit causal inference; therefore, it cannot be concluded that improving MVC or torque steadiness would alter COP behavior during reach-hold tasks. Third, only knee extensor torque characteristics were assessed. The contributions of ankle and hip musculature, trunk control, sensory integration, and multi-joint coordination were not evaluated. Fourth, lower-extremity and trunk kinematics, joint moments, and electromyographic activity were not recorded. Consequently, the movement strategies, muscle activation patterns, and joint-level demands underlying the observed COP behavior could not be identified. Fifth, the reaching foot made light contact with a lightweight block to standardize its position. Although intentional weight bearing was avoided, tactile input or minimal toe contact may have influenced postural behavior. Sixth, the investigators were not fully blinded to participant and task information during data collection and analysis, which may have introduced measurement or analytical bias. Nevertheless, the primary COP and knee extensor torque outcomes were objectively recorded and automatically calculated using standardized procedures. This automated and standardized processing may have reduced, but did not eliminate, the potential influence of investigator-related bias. Seventh, numerous correlations were examined. Although the Holm procedure was used to control the family-wise error rate within the prespecified test families, only one association remained statistically significant after correction. The associations observed only in the unadjusted analyses may represent chance findings and should be interpreted as exploratory. Eighth, torque-steadiness intensities were prescribed relative to each participant’s MVC. The absolute torque corresponding to a given percentage of MVC may be substantially lower in individuals with quadriceps weakness after knee injury than in healthy young adults. Consequently, the neuromuscular demand represented by 70% MVC in the present cohort may not be directly comparable with that in a clinical population, and the present intensity-specific finding should not be directly extrapolated to patients. Future studies should consider both relative contraction intensity and absolute torque demand when comparing healthy and clinical groups. Finally, the primary association was moderate and depended on the operational definition of torque steadiness. Its reproducibility should be examined using different analysis windows and torque-steadiness calculation methods.

Future studies should first determine whether the association between torque CV at 70% MVC and backward reach-hold AP COP path length can be replicated in independent samples. Studies involving individuals after anterior cruciate ligament injury or reconstruction, patients with patellofemoral pain, and other populations with clinically relevant quadriceps dysfunction should evaluate measurement reliability and construct and criterion validity before these assessments are introduced into routine clinical practice. Longitudinal studies are also required to determine whether torque steadiness and reach-hold COP behavior change together during rehabilitation and whether either measure has predictive value for symptoms, functional recovery, reinjury, or return to sport. Incorporating kinematic, kinetic, and electromyographic measurements would further clarify the multi-joint mechanisms underlying forward and backward reach-hold performance.

## Conclusions

In healthy young adults, greater knee extensor torque variability at 70% MVC was moderately associated with greater AP COP movement during backward reach-hold, and this association remained statistically significant after Holm correction. A supplementary linear regression model indicated that torque CV at 70% MVC accounted for 18.9% of the variance in AP COP path length. The other associations observed in the unadjusted analyses did not remain statistically significant after Holm correction and should be regarded as exploratory.

These findings provide preliminary evidence of an association between knee extensor torque regulation at a relatively high testing intensity and AP COP behavior during backward reach-hold. However, they do not establish a direction- or intensity-specific mechanism or the clinical utility of combining torque-steadiness and reach-hold COP assessments. Replication and validation in independent samples and clinical populations are required.
